# Enterectomy for Obstructive Epithelioma at the Ileo-Cecal Valve; Secondary Anastomosis Operation by Abbé’s Long Incision

**Published:** 1893-10

**Authors:** James M. Barton

**Affiliations:** Surgeon to the Jefferson College Hospital and to the Philadelphia Hospital, Philadelphia


### ENTERECTOMY FOR OBSTRUCTIVE EPITHELIOMA AT THE ILEO-CECAL VALVE; SECONDARY ANASTOMOSIS OPERATION BY ABBÉ'S LONG INCISION.

By JAMES M. BARTON, A. M., M. D.,
Surgeon to the Jefferson College Hospital and to the Philadelphia Hospital, Philadelphia.

I saw Mr. B. for the first time on April 18, 1892, at Millville, N. J., in consultation with Drs. Smith and Newell. He was in bed, was very pale and thin, and had frequent attacks of sharp pain while we were talking to him. I obtained the following history: He was twenty-seven years of age, and had been in his usual health until the first of January, when he had an attack similar to the present one lasting one week. From then until the latter part of March he was able to attend to his work in the glass house, but was troubled with some pain and considerable tenesmus. He went to the water closet from four to eight times daily, passing with difficulty a few small scybala each time. His constipation increased until four weeks ago he had several attacks of complete obstruction, each lasting four or five days, and only relieved after taking many purgatives.

He now has, and has had for the last two weeks, about four diarrheic passages daily. He has severe attacks of cramp in the right iliac fossa every few minutes, requiring the constant use of large doses of morphia. These attacks are accompanied by a half inch elevation of the abdominal walls over the painful point. As the pain leaves the abdominal walls descend, and gurgling of wind is heard and felt. He has been confined to his bed for two weeks and is greatly exhausted. There is no elevation of temperature, no tenderness on pressure, and there is no tumor felt, though carefully searched for. There has been no vomiting at any time.

From the absence of vomiting and the marked tenesmus present, I regarded the obstruction as being in or near to the beginning of the large bowel; that it was not far from the ileo-cecal valve was shown by the elevation of the abdominal wall at this point by the obstructed gas. The diarrhea was an evidence of an intussusception, and the very short time the symptoms had existed, and the very complete obstruction present, pointed to a malignant growth as the cause of the intussusception.

Two days later I again visited Millville for the purpose of removing the growth. I was assisted by Drs. Newell and Smith of Millville, Dr. Jones of Camden, and Mr. Borsch, a student at Jefferson College. An incision about three inches long was made in the right iliac fossa, similar to the incision made in removing the appendix. On introducing the finger the growth was found at once. The intussusception could not be reduced, as the epithelioma had grown since it had occurred. After incising the bowel to verify the diagnosis, I removed about six inches of the intestine, including the obstructing epithelioma. Preparations had been made to perform lateral anastomosis at once, but the patient was so severely shocked that the operation was terminated by a temporary artificial anus.

He recovered without difficulty, rapidly gaining flesh and strength, and came to me, in Philadelphia, in June, to have the anastomosis operation performed. I placed him in a private room in the Jefferson College Hospital, and on the 16th I operated, assisted by Drs. Ashton, Fisher, Mr. Borsch, and the house staff. My purpose was to make a long opening, by the method devised by Dr. Abbé, between the lowest possible portion of the ileum and the highest and most convenient portion of the colon; to permit the artificial anus to remain as a safety valve and only close it after the anastomosis opening worked satisfactorily. In order to determine where to make my incision, I probed the bowel at the artificial anus, some days before the operation, with various sounds and catheters, and found it ran directly across the abdomen to the left iliac fossa. As I could not hope to join this to the ascending colon, I decided to use either the transverse colon or the sigmoid flexure, and to open the abdomen by a short incision to the left of the left rectus, as being within reach of both these portions of the large bowel and directly over the portion of the small bowel I wished to use but far enough from the artificial anus to escape the risk of infection. This was an error; the incision should have been made in the median line, rather higher than I did make it and long enough to get a fair view of the intestines.

Just before opening the abdomen I introduced a sound into the artificial anus, so that I might be able to verify, without delay, the portion of the ileum I wished to use after the abdomen was opened.

A three-inch incision was made about two inches to the left of the median line and parallel with it, ending just above Poupart's ligament. The ileum, with the bougie in it, was readily identified, and as the traverse colon hung well into the wound, the sigmoid flexure was not searched for. After stripping the portions of the bowel I intended to use of their contents, and preventing their return by a temporary rubber ligature, the ileum and colon were laid side by side and joined by a line of suture five inches long; when completed the needle was removed and the unused thread permitted to remain. A second line of suture, four and a half inches long, parallel and close to the first was introduced, and the unused threads were also permitted to remain.

Both bowels were then opened by a four-inch incision about a quarter of an inch from the last suture. A third suture was used to join the edges together, passing each stitch across the freshly divided edges so as to check the bleeding. This stitch is known among seamstresses as a "whipping stitch." The pliability of the intestines is such that not only the distal edge can be closed, but a large portion of the edge on the side of the incision toward the operator. A needle was now placed on the unused thread attached to the second line of suture, and this was continued around the ends and in front of the opening, joining the intestines together at about a quarter of an inch from the opening and parallel to it. This suture, when completed, entirely surrounds the opening, and is about a quarter of an inch from it. Lastly, the unused thread of the first suture, which was still hanging at the end, has a needle put on it, and it is carried around the ends and in front of the opening. This suture when completed also surrounds the opening and is about half an inch from it.

The operation can be quite rapidly performed, the intestines being held in contact by an assistant. After the first line of stitching is made it is still easier, and the suture can be made nearly as fast as the same operator would sew a seam in muslin.

The whole operation was performed with a constant stream of tepid boiled water flowing over the intestines we were joining together. As soon as the stitching was completed the intestines were replaced in the abdominal cavity, which was thoroughly flushed, and as the sutured intestines lay in position without undue strain, the abdomen was closed.

The slight vomiting after the ether soon ceased. A number of ounces of fecal matter passed from the artificial anus, but less than before the operation. At the expiration of twenty-four hours there was occasional regurgitation of fluid from the stomach, and when I saw him at 8 o'clock on the second morning, the regurgitated fluids had become coffee-colored. An hour later, forty-three hours after the last operation, I reopened the wound, but failed to find the cause of the obstruction. The sutured intestines lay quite stiffly in place, being kept so by the numerous lines of sutures and the resulting plastic deposit; the small intestine was rather sharply flexed at the extremity, but not enough to obstruct. After as full a search as the condition of the patient warranted, it was concluded that the obstruction must be at the sutured portion. A loop of the ileum above the anastomosis was therefore drawn into the wound and another artificial anus made, but the obstruction was unrelieved, neither gas nor fecal matter passed, and the patient died forty-eight hours later, or four days after the operation.

At the *post-mortem* there was no evidence of recent peritonitis anywhere, the cause of all the difficulty being an old adhesion of the ileum to the abdominal wall at the left iliac fossa. Trailing the ileum up from the original artificial anus in the right iliac fossa, it ran directly across the abdomen to the point of attachment in the left iliac fossa, making a tense band; it then ran downward, and six feet were in the pelvis. I had drawn about two feet of these six under the band and used them for the anastomosis operation. The remaining four feet, still in the pelvis, were sloughing, its circulation being entirely shut off by the band. All the parts above the band including the intestines used in the anastomosis operation, were in admirable condition. The immediate cause of the obstruction was the increased tension of the band, produced by pulling another loop of intestine under it at the operation. This band would probably have given trouble even if the anastomosis operation had not been performed.

The result in this case has not made me feel dissatisfied with the operation. I still think that it is the only tried method of obtaining a sufficiently large opening between intestines. Any smaller opening, no matter how made or with what devices, will be apt to contract and obstruct.

The Abbé operation can be quite rapidly performed. In the case above narrated, the patient suffered from no appreciable shock, though he had but recently recovered from a nearly fatal illness.

In performing this operation again I should make the incision in the median line, and make it higher and longer, so that I might not only see the condition of the intestines, find any abnormal adhesions, but before joining the intestines I should place them in position in the abdominal cavity so that they might lie without strain or tension. The intestines when joined by six lines of sutures are as unyielding as if there were a piece of cardboard between them five inches long. If the intestine does not lie easily in its new position, the bowel at one end or the other of the stiffened portion may be so sharply flexed upon itself as to cause obstruction. In addition, from the same cause, there may be unnecessary strain upon the sutures.

The sutures in the above case were severely tested, the transverse colon was pulled upward by the gastro-colic omentum, while the ileum, a few inches below the suture, was fast anchored by the adhesion. This strain was greatly increased by the subsequent abdominal distention and frequent vomiting. But as the specimen, which I here exhibit, shows, it held perfectly.

The specimen removed at the enterectomy shows almost complete obstruction, the opening that remains being less than a quarterof an inch in diameter.

